# Effectiveness of a mobile-based educational intervention on self-care activities and glycemic control among the elderly with type 2 diabetes in southwest of Iran in 2020

**DOI:** 10.1186/s13690-022-00957-5

**Published:** 2022-09-03

**Authors:** Shahla Vaziri Esferjani, Effat Naghizadeh, Mostafa Albokordi, Mehrnoosh Zakerkish, Marzieh Araban

**Affiliations:** 1 Department of Community Medicine, School of Medicine, Ahvaz Jundishapur University of Medical Sciences, Ahvaz, Iran; 2 Student of Medicine, Student Research Committee, Ahvaz Jundishapur University of Medical Sciences, Ahvaz, Iran; 3 Department of Community Medicine, School of Medicine, Ahvaz Jundishapur University of Medical Sciences, Ahvaz, Iran; 4 Diabetes Research Center, Health Research Institute, Ahvaz Jundishapur University of Medical Sciences, Ahvaz, Iran; 5 Menopause & Andropause Research Center, Ahvaz Jundishapur University of Medical Sciences, Ahvaz, Iran; 6 Department of Health Education and Promotion, Ahvaz Jundishapur University of Medical Sciences, Ahvaz, Iran

**Keywords:** Type 2 diabetes, The elderly, Self-efficacy, Self-care, Attitude, Social support, Randomized controlled trial, Intervention, Education

## Abstract

**Background:**

The elderly constitute a large fraction of patients with type 2 diabetes worldwide. It has been well documented that the elderly’s adherence to disease control is not adequate. The present study aimed to evaluate the impact of a mobile-based educational intervention on self-care behaviors and glycemic control among elderly with type 2 diabetes.

**Methods:**

The present study was conducted on 118 older people (59 in the intervention group and 59 in the control group) with type 2 diabetes who referred to Golestan Hospital in Ahvaz, southwest of Iran in 2020. Participants were randomly divided into experimental and control groups. Data were collected at baseline and after a 3-month follow-up. At baseline, the participants completed a valid and reliable multi-section questionnaire including items on attitude, the multidimensional scale of perceived social support (MSPSS), the Coping Self-Efficacy Scale (CSES), self-care constructs, and HBA1C. After analyzing the pre-test data, we designed a training program which was offered to the intervention group online via mobile phone in three online sessions. The control group, however, received no intervention except diabetes routine care. Data were analyzed using SPSS-15 at a significance level of 0.05.

**Results:**

Before the intervention, the mean scores of CSES, attitudes towards self-care, MSPSS, and self-care were not statistically significant between study groups (*P* > 0.05), but after intervention, the study found significant differences between the groups in terms of CSES, attitude, MSPSS, and self-care (*P* = 0.001). Furthermore, after implementation of the intervention, the mean value of HbA1C in the intervention group was significantly less than that of the control group (7.00 vs. 7.32%) (*P* = 0.001).

**Conclusion:**

The present results indicated that implementing an educational intervention via mobile phone can improve self-care practice and reduce HbA1C in the elderly with type 2 diabetes. The study also showed a moderate to large effect on the outcome variables. However, further studies with longer follow-up periods are recommended to confirm the results.

**Supplementary Information:**

The online version contains supplementary material available at 10.1186/s13690-022-00957-5.

## Highlights

- The study provided a framework for developing a targeted mobile phone health education intervention.

- Mobile phone education can improve both behavioral and clinical indexes. This intervention increased the scores of perceived social support (MSPSS) and Coping Self-Efficacy Scale (CSES), and self-care practice and reduced HbA1C in the elderly with type 2 diabetes.

- Further studies with longer follow-up periods are warranted.

**Supplementary Information:**

The online version contains supplementary material available at 10.1186/s13690-022-00957-5.

## Background

Diabetes as the most common metabolic disease is rapidly increasingaa. It has become a public health concern in the twenty-first century [1, 2]. According to the WHO report, diabetes has been introduced as a serious illness which needs more serious attention from health care providers and physicians [3]. It has been documented that there were approximately 422 million patients with diabetes worldwide in 2020, and most of them were living in low-income countries. In Iran, the prevalence of diabetes was estimated in a national study to be 13.8% in 2013 [2, 4]. However, this figure increases threefold every fifteen years. Based on estimates of the WHO, if special measures are not taken to prevent diabetes, this disease will affect 7 million people in Iran in 2030 [5, 6].

The elderly with diabetes remains a large fraction of the population with diabetes worldwide. One-third of the US elderly population are affected with diabetes [7]. According to one study, 33.6% of the elderly are affected with diabetes in the center of Iran [5]. The same study maintains that while approximately all patients are aware of their disease, their adherence to disease control is not satisfying. Diabetes imposes several costs on health care system and brings about complications such as ischemic heart disease, hypertension, various types of heart failure, retinopathy, neuropathy, nephropathy, and cataract. This medical condition is responsible for 4 million deaths per year and 9% of all deaths worldwide [8–10]. As such, it is reasonable to establish preventive strategies to reduce costs and complications among susceptible groups.

The concept of self-care is an important component of diabetes control and treatment, attracting health policymakers in recent years. This disease requires lifelong self-care behaviors, and its acute and chronic complications can be prevented and delayed by continuous follow-up [11]. In addition to improving the quality of life of individuals and families with chronic diseases, self-care activities play very important roles in reducing treatment costs due to frequent hospitalizations [12]. Studies in the UK indicate that health-promoting self-care behaviors are directly related to the elderly’s health and their quality of life and reduce morbidity and mortality [13].

The effectiveness of providing information and educational strategies to establish self-care behaviors in patients with diabetes has been supported in several studies [2, 14]. Researchers use various methods of distance education and new technologies such as cellphones to create and strengthen the self-care process. Mobile technology in combination with medical specialties has introduced a new possibility known as mobile health which is a mobile-based education, providing new education opportunities [15]. Due to its pervasive nature, this technology provides access to individuals regardless of their geographical and temporal location [16]. Rahnavard et al. (2019) found that mobile-based education was effective in improving self-care behaviors in patients with diabetes [17]. Cui et al. (2016) also reported the beneficial effects of smartphone-based educations on blood glucose control in patients with type 2 diabetes [18].

Considering the increasing number of the elderly with diabetes and the need to empower these patients to do self-care activities, the study aimed to assess the effectiveness of a mobile-based educational intervention on self-care activities and glycemic control among the elderly with type 2 diabetes.

## Methods

The present quasi-experimental study was conducted on the elderly (60 and older) with type 2 diabetes referring to Golestan Hospital in Ahvaz, Iran in 2020 (from June to October). Golestan Clinic is the largest diabetes clinic in Ahvaz, southwest of Iran.

Prior to the commencement of the study, arrangements were made with the Research Deputy of Ahvaz Jundishapur University of Medical Sciences and with officials of the different units of the diabetes clinic of Golestan Hospital of Ahvaz, Iran. Moreover, approval was obtained to conduct the research (Ref. ID: IR.AJUMS.MEDICINE.REC.1399.014).

### Eligibility criteria for participants

Inclusion criteria were: a minimum age of 60 and higher, ability to communicate in Persian, no cognitive problems (minimum score of the short-form cognitive test > 6), minimum literacy, not living in a nursing home, having cellphone to receive educational contents, and willingness to participate in the study. Participants who were not willing to continue participation in the study or were inaccessible due to changes in their address and phone number, as well as those who did not attend the educational sessions were excluded from the study.

### Sample size

Assuming a confidence interval of 95%, error of 5%, and test power of 80% and based on the findings of a similar study [28], sample size was 55 for each group. Assuming a 15% probable attrition rate, we finally selected a total of 126 eligible individuals from patients' file numbers and included them in the study.

### Randomization

Randomization was achieved using sealed, opaque, sequentially numbered envelopes prepared using a random number generator. A research assistant who was not involved in the recruitment of participants prepared the envelopes. Participants allocated to the control group (63) received standard care whereas participants assigned to the intervention group (63) received a mobile phone intervention program in addition to their routine care. All participants completed a follow-up questionnaire at a 3-month follow-up with a research assistant blinded to group allocation.

### Measures

The data collection tool was a multi-section questionnaire including the following sections:

1. Demographic medical information sheets containing information about age, sex, education level, marital status, occupation, height, weight, duration of illness, levels of HBA1C, and sources of information about the disease.
2. Self-care questionnaire by Glasgow & Toobert [19]: The questionnaire included 11 questions measuring patients' self-care behaviors in the last 7 days with questions such as "How much did you control your blood glucose during the last week?" Questions included information about blood glucose testing (2 questions), exercise self-care (2 questions), foot self-care (2 questions), diet adherence self-care (4 questions), and smoking cessation self-care (1 question). Each behavior had a variable score from 1 to 7 and its final score was from 7 to 77. The reliability of the scale was confirmed with a Cronbach's alpha of 0.78, which was satisfactory.
3. The Coping Self-Efficacy Scale(CSES): This questionnaire included 13 items such as "I try to pray to remain calm when I am under pressure". The answer to each question was set on a 10-point Likert scale from Never = 0 to I Can = 10. The scores range from 0 to 130. The validity and reliability of the Persian version of the questionnaire were confirmed in a study in Iran [4] where the internal consistency of the questionnaire was confirmed by obtaining a Cronbach's alpha of 0.91.
4. The multidimensional scale of perceived social support (MSPSS): The scale consisted of 12 items such as "I have friends with whom I can share happiness and sadness ", and it evaluated the perceived support of a person by friends, family, and others [20]. The answers were set on a 7-point Likert scale from 0 to 6 (strongly disagree to strongly agree, respectively). The range of achievable points was between 0 and 72. The reliability of the questionnaire was confirmed by obtaining a Cronbach's alpha (0.85) in our study.
5. The attitude towards self-care questionnaire: It included 14 questions such as "regular sports (walking and exercise) were effective in controlling my diabetes". The answers were set on a 5-point Likert scale from strongly disagree with a score of 1 to strongly agree with a score of 5. The scale yielded a score range from 14–70, with higher scores indicating better results. The reliability of the questionnaire was confirmed by Karimy et al. [4]. The reliability of the questionnaire was confirmed by obtaining a Cronbach's alpha (0.92) in our study.

### Procedure

At the diabetes clinic of Golestan Hospital, Ahvaz, southwest of Iran, a list of potentially eligible patients who were willing to participate in the study was generated by a last-year medical student. A research assistant approached the potential participants and provided them with details about the study. After informed consent was obtained from the participants, the participants were randomized to intervention and control groups, and baseline data were collected Two cell phone numbers were obtained from each participant for delivery of educational contents and follow-up. Participants were asked to briefly confirm that the contents had been received. If researchers did not receive this confirmation, a member of the research team would call, or send educational contents to the other cell phone number. Moreover, the participants were asked to introduce one of their relatives to the instructor in order to receive educational programs and help the patient. Also, the participants were added to Wattpad groups created by the research team so that the participants could receive the materials which taught in online classes and/or discuss the self-care practices with each other. Data were gathered at baseline and after a 3-month follow-up to examine the effects of education on the study outcomes.

### Intervention

After completing the pre-test questionnaires in the control and intervention groups and measuring and recording the patients' HbA1c levels, we designed an educational program (Additional file 1; The educational program content provided for the elderly with type 2 diabetes in southwestern Iran, 2020). We designed and developed the educational content based on coping self-efficacy, attitude, and social support. We validated the educational program content according to Harrison's *Endocrinology* and *Infectious Diseases* (Standards of Medical care in diabetes, 2020) and the "Up To Date" website. Furthermore, three health education experts, two internal medicine specialists, and an IT expert with health informatics expertise approved the program content. The intervention was implemented by holding three training sessions indirectly (online and using WhatsApp) for the experimental group. Each training session lasted 60 min. These online sessions were held in groups of 10–11 participants. It should be noted that the intervention sessions were held every other day for a total of 9 days to encourage patients in the experimental group to continue participating in the intervention, prevent them from forgetting the content, and follow up the patients in terms of their attention to the content. To create social support, the patients were asked to talk about their problems with the disease to the researcher and other patients in the WhatsApp group at the end of each training session. This was done so that essential measures could be taken and plans could be made to solve their problems if necessary. To increase coping self-efficacy, we focused on the antecedents (e.g., sources of information) that may be used to influence self-efficacy for coping with problems associated with self-care practices, performance accomplishment (e.g., past experience in doing self-care activities), and verbal persuasion (e.g., encouragement and support from researchers and others). To improve attitude towards self-care, an informative booklet was prepared by a health education specialist and validated by a panel of experts, and it included written text (short messages about diabetes and its complications) with illustrations to reinforce the information needed to do self-care practices and prevent complications. In online sessions, the patients were asked to compare and discuss other patients' levels of health with and without doing self-care activities. The control group received routine care from the health center. It is noteworthy that standard care in Iran includes every three months visits at a healthcare clinic by a doctor, a dietitian, and a nurse, and each visit lasts for less than 20 min. These visits were held individually or in groups.

### Statistical analysis

All data analyses were conducted according to a pre-established analysis plan using SPSS 15 (SPSS, Inc., Chicago, IL, USA). First, the normality of the scores was evaluated and measured, using the Kolmogorov–Smirnov test. The proportions were compared using the chi-square test and Fisher's exact test. The independent t-test was used to compare two groups in terms of age and duration of disease. To assess the effect of the intervention on outcome variables, ANCOVA was performed adjusting for baseline pre-intervention outcomes and potential covariates (gender, age, type of medication). The significance level was set at 0.05.

## Results

**Fig. 1:**
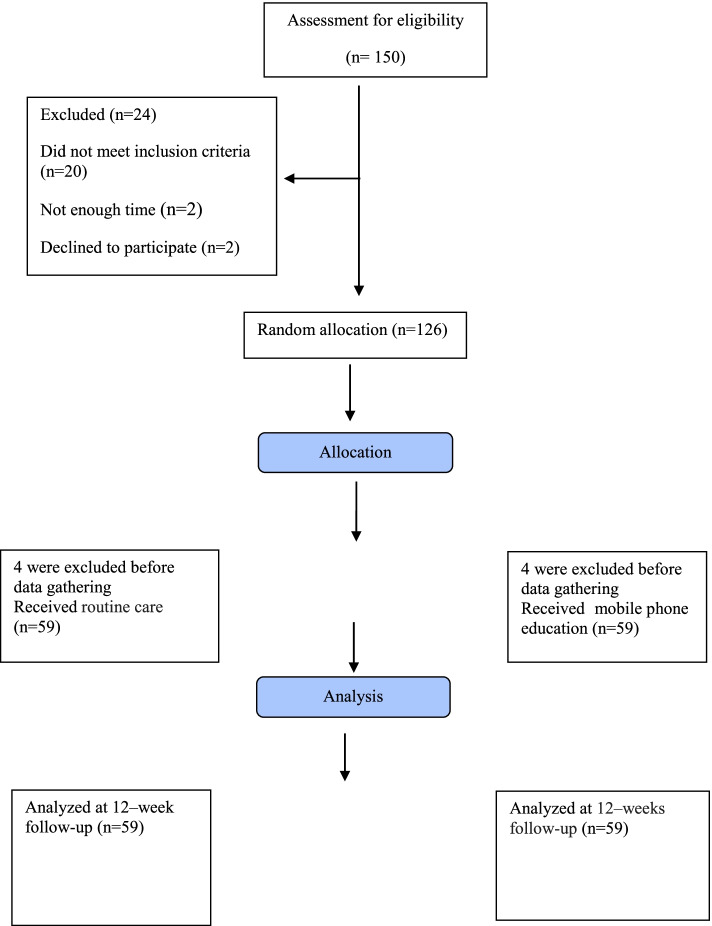
Flow diagram of the study among patients with type 2 diabetes over the year 2020 in south west of Iran

In this study, of all 150 patients who were initially assessed, 20 were excluded due to not meeting the inclusion criteria. Two patients withdrew from the study, and two stated that they did not have enough time to participate in the study. Also, before the study starts, 8 patients (4 in the experimental group and 4 in the control group) were excluded from the study due to leaving wrong phone numbers. The study started with 118 patients in the two groups of intervention and control, and the final analysis was performed on data obtained from 118 patients (59 in the intervention group and 59 in the control group). Figure 1 shows the flow diagram of the study.

The participants' mean age was 63.5 ± 2.26 years, with an age range between 60–70 years. In terms of gender, 54 (45.8%) were male and 64 (54.2%) were female. Our results indicated that the experimental and control groups were similar in terms of age, gender, education level, ethnicity, marital status, lifestyle, income level, type of medication, and duration of illness (*P* > 0.05). Moreover, there was no statistically significant difference between the patients in the two groups at baseline (Table 1).

**Table 1:** Baseline characteristics of the elderly with type 2 diabetes in both intervention and control groups in south west of Iran in 2020

Group		Intervention (*n* = 59)	Control (*n* = 59)	*P*-value
Variable				
Age (year)	60–64	37(62.7%)	40(67.8)	0.562^a^
	65–70	22(37.3%)	19(32.2%)	
Gender	Male	26 (44.1%)	28 (47.5%)	0.712^a^
	Female	33 (55.9%)	31 (52.5%)	
Marital status	Single	13 (22%)	16 (27.1%)	0.521^a^
	Married	46 (78%)	43 (72.9%)	
Education level	Illiterate	2 (44.1%)	31(52.5%)	0.357^a^
	Under high school diploma	26 (44.1%)	25 (42.4%)	
	High school diploma and higher	7 (11.9%)	3 (5.1%)	
Ethnicity	Fars	32 (54.2%)	38 (64.4%)	0.261^a^
	Non-Fars	27 (45.8%)	21 (35.6%)	
Lifestyle	Without spouse	5 (8.5%)	8 (13.6%)	0.378^a^
	With spouse	54 (91.5%)	51 (86.4%)	
Income level (million Tomans per month)	Less than 2	38 (64.4%)	43 (72.9%)	0.577^a^
	2 to 5	17 (28.8%)	12 (20.3%)	
	Higher than 5	4 (6.8%)	4 (6.8%)	
Type of medication	Regular	25(42.4%)	22(37.3%)	0.10^a^
	NPH	13(22.2%)	10(17%)	
	Glargine	18(30.5%)	19(35.54)	
	Pill	3(5.1%)	6(10.16%)	
Duration of diabetes (Years)		5.80 ± 2.469	5.68 ± 3.406	0.507^b^

^a^ Derived from chi-square

^b^ Derived from t test

### Results of outcome measures

#### Self-care

The mean score of self-care was not statistically significant in the patients in the two groups before intervention (*P* > 0.05). However, it was significantly higher in the intervention group compared with the control group after intervention (53.56 vs. 44.95) (*P* < 0.001). In other words, the mean of self-care changes was significant in patients in the intervention group (8.74 units of increase) (*P* < 0.001). However, there were no significant changes in the control group (*P* > 0.05). (Table 2).

**Table 2:** Comparison of constructs of interest among the elderly with type 2 diabetes at baseline and after a 3-month follow-up in south west of Iran in 2020

*Outcome*	*Baseline*	Follow-up	*P*-value*	*P*-value^£^
***Coping Self-efficacy***				
Experimental group	70.93 ± 24.50	84.49 ± 24.40	0.001	0.002
Control group	69.58 ± 25.34	70.25 ± 23.25	0.67	
*P*-value**	0.75	0.001	-	
***Attitude towards self-care***				
Experimental group	36.85 ± 12.80	48.66 ± 13.90	0.001	< 0.0001
Control group	34.22 ± 12.53	37.76 ± 10.27	0.59	
*P*-value**	0.24	0.001	-	
***Social support***				
Experimental group	44.59 ± 16.19	54.44 ± 16.08	0.001	< 0.0001
Control group	43.42 ± 16.20	44.47 ± 15.76	0.08	
*P*-value**	0.64	0.001	-	
***Self-care***				
Experimental group	44.81 ± 17.21	53.56 ± 13.32	0.001	0.001
Control group	43.32 ± 15.37	44.95 ± 11.60	0.15	
*P*-value**	0.68	0.001	**-**	

Values are presented as mean ± SD

^*^ Paired T-test

^**^ Two independent samples T-test

^£^ Adjusted for baseline Pre-intervention outcomes and potential covariates (gender, age, type of medication)

### Coping self-efficacy scale

The mean score of coping self-efficacy was not significantly different in the patients of the two groups before intervention (*P* > 0.05). However, it was significantly higher in the experimental group compared with the control group after intervention (*P* = 0.001). In other words, the mean changes in coping self-efficacy (13.5 units of increase) were statistically significant in patients in the intervention group (*P* < 0.001). However, there were no significant changes in this regard in the control group (*P* > 0.05) (Table 2).

#### The multidimensional scale of perceived social support (MSPSS)

The mean score of MSPSS was not significantly different in the patients of the two groups before intervention (*P* > 0.05). However, this score was significantly higher in the experimental group compared with the control group after intervention (*P* = 0.001). In other words, the mean changes in social support (9.8 units of increase) were statistically significant in patients in the intervention group (*P* < 0.001), while no significant changes were observed in the control group (*P* > 0.05) (Table 2).

### Attitude towards self-care

The mean score of *attitudes towards self-care* was not significantly different in the patients of the two groups before intervention (*P* > 0.05). However, it was significantly higher in the experimental group compared with the control group after intervention (*P* = 0.001). In other words, the mean changes in attitudes towards self-care (11.8 units of increase) were statistically significant in patients in the intervention group (*P* < 0.001). However, no significant changes were observed in the control group (*P* > 0.05) (Table 2).

#### HBA1C

According to Table 3, the mean HbA1C score was not significantly different in the patients of the two groups before intervention (*P* > 0.05). Nevertheless, it was significantly lower in the intervention group compared with the control group after education (7.00 vs. 7.32%) (*P* = 0.001). In other words, the mean changes in patients' HbA1C were statistically significant in the intervention group (0.22 units of decrease) (*P* < 0.001) whereas no such significant changes were observed in the control group (*P* > 0.05).

**Table 3:** Comparison of glycosylated hemoglobin (HbA1c) levels among the elderly with type 2 diabetes at baseline and after a 3-month follow-up in south west of Iran in 2020

*Outcome*	*Baseline*	Follow-up	*P*-value*	*P*-value^£^
***HbA1c***				
Experimental group	7.23 ± 0.48	7.00 ± 0.46	0.001	0.0001
Control group	7.33 ± 0.63	7.32 ± 0.58	0.97	
*P*-value**	0.63	0.001	-	

Values are presented as mean ± SD

^*^ Paired T-test

^**^ Two independent samples T-test

^£^ Adjusted for baseline Pre-intervention outcomes and potential covariates (gender, age, type of medication)

## Discussion

The present study aimed to investigate the effect of a mobile-based education program on the self-care of older people with type 2 diabetes. The results indicated that this program increased the mean score of self-care and significantly reduced the patients' blood glucose. This was consistent with Martos-Cabrera et al. who found that smartphone apps could help patients with diabetes improve their HbA1c levels [21]. In another similar study, McMahon et al. reported that patients receiving online education had a significant difference in controlling blood glucose indices compared to patients receiving the traditional education [22]. Norris et al. studied blood glucose management and control in adults with diabetes and found that the implementation of electronic self-care training programs was a good way to educate the patients due to increased patient participation in self-care activities [23]. In a meta-analysis on diabetes management and blood sugar control, Welch et al. confirmed the need for virtual educational services to help all patients [24]. Another meta-analysis by Aminuddin also indicated that smartphone-based interventions had beneficial effects on self-care activities and health outcomes in patients with type 2 diabetes mellitus (T2DM) [25]. However, further research is required to evaluate the effectiveness of smartphone-based self-care interventions for T2DM. According to previous research, successful control of diabetes through proper self-care performance seems to depend on large-scale and organized patient training tailored to their educational requirements and other conditions. Hence, proper educational interventions and encouraging patients to do self-care behaviors can largely prevent or at least delay the acute and chronic complications of the disease.

According to our results, the patients' mean score of coping self-efficacy in the experimental group had a statistically significant increase after the educational intervention, which is consistent with similar studies in this field [2, 26]. Previous studies support the role of self-efficacy as an important construct in the development of self-care behavior [27–31]. For instance, Amer et al. indicated that self-efficacy was significantly associated with adherence to self-care activities and blood sugar control [32]. In another similar study by Ahmad et al. in Saudi Arabia [33], self-efficacy significantly predicted the self-management of some behaviors. They found that patients with a greater understanding of and belief in self-efficacy had better self-management behaviors when it comes to following a diet, doing exercise, testing blood sugar, and doing foot care activities. This finding helps existing research focus on improving self-efficacy as a key construct to improve diabetes self-care.

Based on our findings, creating a WhatsApp education group and sending a designed education program to the patient's acquaintances had a positive effect on creating perceived social support in these patients. Given the role of social support in creating self-care behavior in chronic diseases, which has already confirmed in previous studies [4, 12], health care providers should evaluate patients with diabetes and their family members to develop different types of social support and improve the patients' self-care. In this regard, the results of a study indicated that more social support from family members and relatives was associated with better diabetes self-care among patients with T1DM and T2DM [34].

The results of the present study indicated a significant increase in the mean score of attitudes in patients in the experimental group after the educational intervention. The discussion method adopted by the educator and presentation of positive and negative experiences of patients made the participants more willing to use self-care behaviors through the modeling process. In fact, after an individual directly experiences a behavior, positive beliefs about the consequences of that behavior are reinforced while the person’s motivation is still maintained [4]. Consistent with our findings, Plotnikoff et al. [35] and Zhong et al. [36] also confirmed the role of attitude in the higher adherence of patients with diabetes to physical activity. In a study by Omondi on Kenyan patients with diabetes, attitude was the strongest predictor of dietary behavior [34]. The finding was important since based on previous studies, a positive attitude towards a particular behavior has been proved to lead to a greater willingness to engage in that behavior.

Our study showed a moderate to large effect of the intervention on the outcome variables. This is important given the increasing number of the elderly population, and the need for empowering them in doing self-care practices at home and reducing their referral to medical centers.

### Research limitations

First, minimum literacy as an inclusion criterion in this study may make it difficult to generalize the results to all patients in society. Also, the study examined only people over 60 years of age with type-2 diabetes in the clinic of Golestan Hospital, Ahvaz, Iran. Hence, the results cannot be generalized to people with type-1 diabetes, pregnant mothers with gestational diabetes, and people with type-2 diabetes under the age of 60. Therefore, we suggest conducting similar studies dedicated to these populations in different cities.

## Conclusion

The present results indicated that implementing an educational intervention via mobile phone can improve self-care practices and reduce HbA1C in the elderly with type 2 diabetes. The study also showed a moderate to large effect on the outcome variables. Further studies with longer follow-up periods are recommended to confirm our results.

## Supplementary Information

**Additional file 1.** The educational program content provided for the elderly with type 2 diabetes in south western Iran, 2020.

## Data Availability

Upon reasonable request, onsite (Ahvaz Jundishapur University of Medical Sciences, Ahvaz, Iran) access to the data can be provided.

## Abbreviations

CSES: Coping Self-Efficacy Scale
MSPSS: Multidimensional scale of perceived social support
