# Supplementary material for: Effectiveness of a mobile-based educational intervention on self-care activities and glycemic control among the elderly with type 2 diabetes in southwest of Iran in 2020
Source: Arch Public Health. 2022 Sep 3;80:201. doi: 10.1186/s13690-022-00957-5 (PMC9441036; doi:10.1186/s13690-022-00957-5)
Supplement: Supplementary file 1 — Additional file 1. The educational program content provided for the elderly with type 2 diabetes in south western Iran, 2020. [file 13690_2022_957_MOESM1_ESM.docx]

Additional file 1: The educational program content provided for the elderly with type 2 diabetes in south western Iran, 2020

Topics taught to patients in each session included the following

Reciting a short surah and a hadith on health issues

Defining old age and related issues in three one-minute audio files

Defining the disease, risk factors and development of type 2 diabetes along with an instructional video

Dedicating 5 minutes of question-and-answer in relation to these topics to encourage patients to continue the intervention.

Explaining the complications of the disease (foot ulcers and other parts of the body, neuropathy, nephropathy, retinopathy, heart attacks, and strokes) and allocating 15 minutes of question-and-answer to all patients in the group about the presence or absence of any of the above complications in them, with the aim of identifying and examining the complications, and referring the patients rapidly for treatment and prevention of other complications.

Explaining all the content provided in audio and PDF formats for easier access to topics for summary and retention

Understanding the nature, progress, complications and prognosis of type 2 diabetes

Prevention

Control

Treatment

Dedicating the first 10 minutes of the session to the previous discussion

Dedicating the last 10 minutes to question-and-answer about the session contents

The importance of self-care and achieving a clear understanding of the consequences of neglecting it, the benefits and costs of self-care and self-efficacy, and the effect of strengthening self-care on the control and treatment of type 2 diabetes (audio descriptions were provided to the groups)

Setting behavioral goals and planning to change the behaviors that are needed to create new behaviors

The most important behaviors expected from the patient are listed in the table on the next page

| 1 | - - A nutritionist should be consulted for a proper diet, limiting the consumption of fried foods and sweets, and promoting the consumption of fresh fruits and vegetables. - A regular exercise program should be devised. (Overweight and obese people should walk for at least 30 minutes a day.) - - Smoking and alcohol consumption should be stopped. - - Stress and excitement should be reduced by visiting a psychiatrist, doing exercise, and paying attention to spiritual matters, - Disease-related variables such as blood sugar, weight, and blood pressure should be measured on a regular basis. |
| --- | --- |
| 2 | - - Foot ulcers should be prevented by wearing strong cotton socks and standard medical shoes (corresponding foot size, being soft and comfortable, and having strong sides to protect the foot). - - Slippers should be used for long walks, and foot hygiene should be observed through regular foot washing - - A patient's family member should be responsible for regulating the water temperature in order to prevent burns caused when the patient does not feel the temperature of objects and liquids due to diabetic neuropathy, or before the patient uses hot water for bathing or other activities in which hot water is used. - - The use of any hot object should be avoided. - - The extremities of the body, such as the lower limbs and genitals, should be examined by the patient to prevent ulcers, and if the patient is incapacitated, the patient's caregivers should do this. - - To treat any wound, especially in the extremities of the body, infectious and internal medicine specialists should be referred to as soon as possible and any self-medication and neglecting chronic wound healing should be avoided. - Oral hygiene should be observed through regular brushing and disinfection of all surfaces of the mouth using mouthwash. |
| 3 | - Community resources that help the patient achieve care goals should be identified and communicated with. |
| 4 | - - Medications should be taken regularly. - A nephrologist and an ophthalmologist should be regularly visited in order to prevent or treat minor vascular diseases. |

The points that were emphasized to the patient's companions:

| - - One of the patient's caregivers should be responsible for reminding the patient and helping him/her to take the medicine regularly. In order to take the medicine on time, the patient's cell phone alarm can be used so that the patient can take the medicine correctly according to the doctor's instructions. - - To prevent burns caused by hot objects and liquids in women with type 2 diabetes who were able to cook, the patient was cautioned to avoid lifting heavy and hot objects (e.g., metal dishes, samovars, etc.), or having any contact with similar items. This was later emphasized to the patient's companion too. - - In order to encourage and motivate the participants, the patient's companions were asked to accompany the patient in walking or other physical activities such as public sports, as much as possible. In addition to increasing motivation and self-confidence in the elderly with diabetes, this reassures the patient's companions to monitor the patient in terms of adherence to regular activity and prevent injury to patients during physical activity. - - In the elderly over 70 years of age with diabetes, in no case should the patient be left alone at home or out of the residence for a long time, and if possible, a family member or caregiver should take care of him/her.   . |
| --- |

When patients visit their physician, one of their caregivers should be with them in order to write down the doctor's recommendations, especially the changes in dosage or timing of medications, and the handwriting should be legible so that it can be later used by the patient.

The amount of insulin the patient receives should be written down so that the patient is reminded of it at each meal.

- The patient's glucose measuring devices (the measuring device, the needles, and the kits for measuring glucose) should be checked on a weekly basis in order to ensure the health and sterility of these devices.

- The amount of calories received daily, the amount and type of physical activity, the time required to perform it, the intervals of using drugs, and the time of measuring blood glucose as a daily schedule should be recorded and negotiated with the patient in order for him/her to adhere to it.
